# Methods for merging data sets in electron cryo-microscopy

**DOI:** 10.1107/S2059798319010519

**Published:** 2019-08-23

**Authors:** Max E. Wilkinson, Ananthanarayanan Kumar, Ana Casañal

**Affiliations:** a MRC Laboratory of Molecular Biology, Francis Crick Avenue, Cambridge Biomedical Campus, Cambridge CB2 0QH, England

**Keywords:** cryo-EM, merging of data, single-particle processing, *RELION*

## Abstract

Recent developments have resulted in electron cryo-microscopy (cryo-EM) becoming a useful tool for the structure determination of biological macromolecules. For samples containing inherent flexibility, heterogeneity or preferred orientation, the collection of extensive cryo-EM data using several conditions and microscopes is often required. In such a scenario, merging cryo-EM data sets is advantageous because it allows improved three-dimensional reconstructions to be obtained. Since data sets are not always collected with the same pixel size, merging data can be challenging. Here, two methods to combine cryo-EM data are described. Both involve the calculation of a rescaling factor from independent data sets. The effects of errors in the scaling factor on the results of data merging are also estimated. The methods described here provide a guideline for cryo-EM users who wish to combine data sets from the same type of microscope and detector.

## Abstract 2

A workflow to combine cryo-EM data collected at different magnification.

## 1. Introduction   

Over the last decade, electron cryo-microscopy (cryo-EM) has become a powerful tool to resolve three-dimensional (3D) structures of biological specimens at a resolution sufficient for proposing *de novo* atomic models (Kühlbrandt, 2014 ▸; Smith & Rubinstein, 2014 ▸; Cheng *et al.*, 2017 ▸). This has primarily been possible through progress made in the development of direct electron detectors (Battaglia *et al.*, 2009 ▸; Faruqi & McMullan, 2011 ▸; Li *et al.*, 2013 ▸; McMullan *et al.*, 2014 ▸) and improvements in image-processing algorithms (Scheres & Carazo, 2009 ▸; Scheres, 2012 ▸). The result of these advances is a rapid growth in the number of cryo-EM structures deposited per year in the Electron Microscopy Database (EMDB; https://www.ebi.ac.uk/pdbe/emdb/statistics_mol_wt.html/, https://www.ebi.ac.uk/pdbe/emdb/statistics_min_res.html/).

Cryo-EM can be performed on small amounts of biological samples (in the microlitre to femtolitre range; Russo & Passmore, 2016 ▸; Ashtiani *et al.*, 2018 ▸; Noble, Wei *et al.*, 2018 ▸) in a relatively short time (from hours to days; Kimanius *et al.*, 2016 ▸; Cianfrocco *et al.*, 2018 ▸). A suitable sample for cryo-EM is homogenous and evenly dispersed in random orientations throughout the support holes. However, many biological specimens are challenging targets that possess intrinsic flexibility, conformational heterogeneity or adopt a preferred orientation in the vitreous layer of ice (Nogales *et al.*, 2016 ▸; Plaschka *et al.*, 2017 ▸). Such problems can be reduced by optimizing the biochemical preparation of the sample, changing the type and chemistry of the support grids (Meyerson *et al.*, 2014 ▸; Russo & Passmore, 2014 ▸; Boland *et al.*, 2017 ▸; Liu *et al.*, 2019 ▸) and using different detergents (Takizawa *et al.*, 2017 ▸; Drulyte *et al.*, 2018 ▸; Chen *et al.*, 2019 ▸). All of the above can lead to the necessity to collect data in various conditions. For low-concentration samples, or when inherent heterogeneity or preferred particle orientation is difficult to eliminate, collecting a large amount of cryo-EM data is a valid option. For example, the number of particles with rare views might be enriched in large data sets, as distribution and orientation can highly depend on the ice thickness (Casañal *et al.*, 2017 ▸; Noble, Dandey *et al.*, 2018 ▸). Also, large data sets can allow extensive classification to computationally isolate subpopulations of particles in different states (Fernández *et al.*, 2013 ▸; Urnavicius *et al.*, 2015 ▸; Matzov *et al.*, 2017 ▸; Wilkinson *et al.*, 2018 ▸; Charenton *et al.*, 2019 ▸; Guo *et al.*, 2019 ▸).

**Figure 1:**
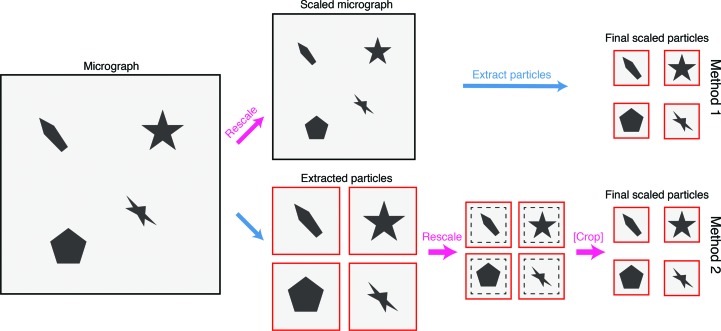
Schematic overview of two methods for scaling cryo-EM data. In Method 1, micrographs are scaled (pink arrow) before particles are extracted (blue arrow). In Method 2, particles are extracted and then scaled, with a optional cropping step if required.

There are many practical limitations when it comes to collecting and processing such large cryo-EM data sets. The lack of availability of ample microscope time within an institute or cryo-EM facility means that users often collect several data sets with different microscopes, either within their host institute or across the world in dedicated cryo-EM facilities (Casañal *et al.*, 2017 ▸; Menny *et al.*, 2018 ▸; Fica *et al.*, 2019 ▸). Also, when microscopes are under repair or upgraded (for example the incorporation of a new detector or energy filter), or when changing the magnification for data collection is needed (for example increasing the resolution or the number of particles per image), users might collect at different pixel sizes. The absence of a streamlined procedure to combine cryo-EM data that differ in pixel size poses an additional challenge in structure determination in single-particle projects. Here, we describe two straightforward methods to combine cryo-EM data sets (Figs. 1 ▸ and 2 ▸). We present two case studies. In the first, data sets were collected using two distinct Titan Krios microscopes with similar magnifications. In the second, data acquisition was performed on the same Titan Krios microscope at different magnifications.

## 2. Procedure   

### 2.1. General considerations   

Before combining data sets, it is essential to take into account two primary considerations. Firstly, the relative pixel size (Å per pixel) between data sets needs to be accurately determined. One data set must be rescaled to match the other (the reference) such that the particles can be aligned to the same 3D reference. The success of this process will depend on how accurately the relative pixel sizes are estimated. Secondly, the absolute pixel size of the reference data set should be determined precisely. Accurate knowledge of the absolute pixel size is required for defocus determination, contrast transfer function (CTF) correction and map interpretation (Wade, 1992 ▸; Cheng *et al.*, 2015 ▸). The exact absolute pixel size often deviates from the nominal pixel size owing to slight differences in the optics and position of the detectors between different microscopes of the same type. Ideally, one could determine the absolute pixel size for both data sets before or during data collection. This can be performed using methods such as cross-grating grids (lower magnification regime), titanium dioxide (medium magnification regime) and gold on grids in the form of gold particles or gold foil (higher magnification regime) (Cheng *et al.*, 2015 ▸). UltraAufoil grids are particularly useful as the reflections of the gold on the grid can be used to calibrate the pixel size under the same imaging conditions as used for data collection (Russo & Passmore, 2014 ▸; Cheng *et al.*, 2015 ▸). If pixel sizes are accurately determined before data processing one can rescale the different sets of micrographs to have the same pixel size and treat the data sets as one.

However, in many cases users rely on the nominal pixel size given by the facility and might not be able to determine the exact pixel size after their microscope session has ended, or the absolute pixel sizes might have been measured inaccurately. For this, we present an empirical approach to determine the pixel size of one data set relative to the nominal pixel size of the reference data set. Even if the exact pixel size is known, this method can be used to confirm the relative pixel size between data sets.

### 2.2. Determination of the scaling factor between data sets by cross-correlation   

In principle, determination of the scaling factor between data sets by cross-correlation is straightforward. All that is needed are independent 3D reconstructions for each data set. By cross-correlating the maps at different relative pixel sizes it is possible to determine their scaling factor. To illustrate this concept (and later demonstrate Method 1; Section 2.3.1), two different data sets of the polymerase module of the cleavage and polyadenylation factor (CPF) from *Saccharomyces cerevisiae* are used, collected at nominal pixel sizes of 1.40 Å per pixel (data set I, Krios 2 at MRC-LMB) and 1.36 Å per pixel [data set II, Diamond Light Source (DLS) electron Bio-Imaging Centre (eBIC)] (Casañal *et al.*, 2017 ▸). To calculate the relative pixel size and scale the data, the pixel size of the data set with the larger pixel size (1.40 Å per pixel; reference; data set I in this example) should be kept constant, while changing the relative pixel size of the second data set (1.36 Å per pixel; data set II).

#### 2.2.1. Obtain 3D reconstructions from each data set   

**Figure 2:**
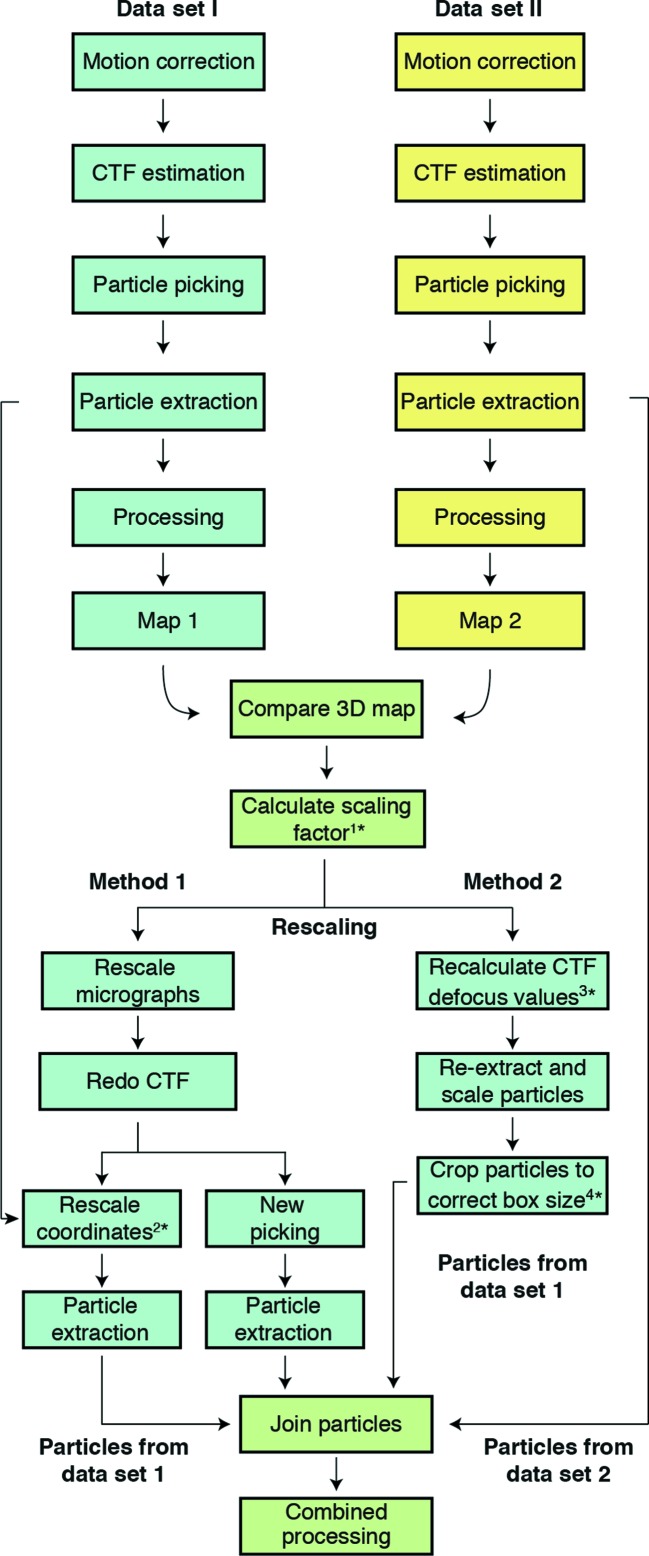
Flowchart of the data-merging process. The first step for both methods is obtaining independent 3D reconstructions to calculate the scaling factor between data sets. Method 1 rescales the data at the level of micrographs and Method 2 uses extracted particles. It is essential to redo the CTF estimation (Method 1) or to apply the scaling factor to defocus values to recalculate the CTF (Method 2). Once the particles from the two data sets have been merged (‘Join’ job in *RELION*), further processing can be carried out using standard procedures. Asterisks (*) indicate where scripts are provided to perform different steps in the process: 1*, determine_relative_pixel_size.py; 2*, rescale_particles.py; 3*, scale_ctf.sh; 4*, boxscaler.py.

In *RELION*, the processing of individual data sets involves motion correction, CTF estimation, particle picking, extraction, several classification steps and refinement (Scheres, 2012 ▸; Fernandez-Leiro & Scheres, 2017 ▸). More extensive processing can be performed, including Bayesian polishing and CTF refinement (Zivanov *et al.*, 2018 ▸, 2019 ▸). However, these additional steps are not usually required before rescaling data sets (Fig. 2 ▸). Nevertheless, it is desirable to obtain high-resolution 3D reconstructions of individual data sets, as the resolution of the maps is directly linked to the accuracy of the scaling factor. In most cases, 4–5 Å resolution is sufficient. If large flexible areas are present, the comparison of maps can be improved by masking.

#### 2.2.2. Calculate and optimize the scaling factor between 3D maps   

**Figure 3:**
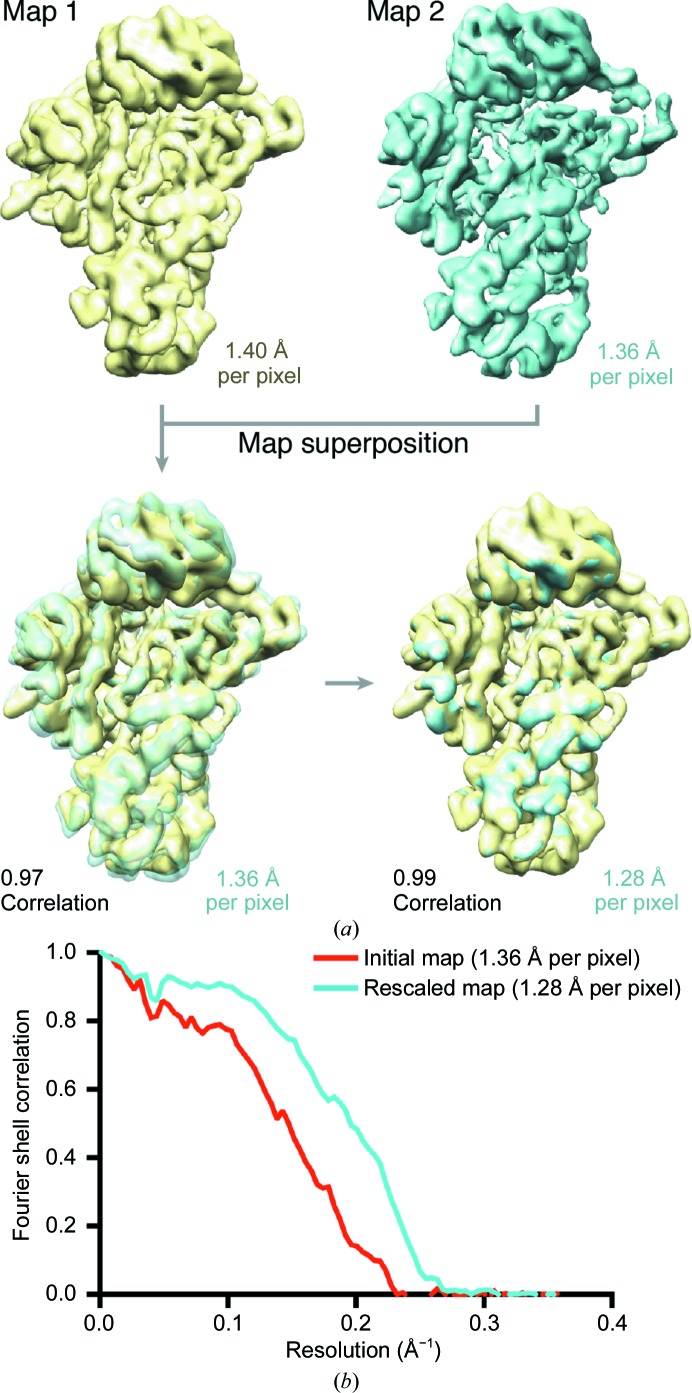
Scaling factor between data sets. (*a*) Independent 3D maps obtained from data set I (yellow) and data set II (cyan) vary in volume size owing to differences in the nominal pixel sizes. Superposition of such maps in *Chimera* shows that correlation between the reconstructions improves when the pixel size of map 2 (cyan) is scaled to fit the pixel size of map 1 (yellow). (*b*) Fourier shell correlation (FSC) between maps 1 and 2, before and after scaling. The correlation increases at 1.28 Å per pixel.

Real-space correlation is recommended to compare the volumes of independently calculated maps. Cross-correlation in real space can be determined, for example, using *Chimera* (Pettersen *et al.*, 2004 ▸), where superposition and fitting of the maps can be achieved using the ‘Fit in Map’ command [Fig. 3 ▸(*a*)]. This tool provides a correlation coefficient between the maps that indicates the quality of the fitting. The Volume Viewer tool can be used to adjust the pixel size, using real-space interpolation, of the target map [data set II, map 2, Fig. 3 ▸(*a*)] by entering an adjusted voxel size into ‘Features’, ‘Coordinates’.

Also, we provide a Python script (https://www.python.org) called determine_relative_pixel_size.py (Supplementary Script 1) that uses *relion_image_handler* (Scheres, 2012 ▸) and the Fourier shell correlation (FSC; Harauz & van Heel, 1986 ▸) to determine an optimal pixel size for the target map. The output is a pixel size range in which the FSC remains the same. This script uses the files map1.mrc and map2.mrc and their corresponding pixel sizes as input, carries out rescaling of map 2 to a set of pixel sizes and measures cross-correlation between the two 3D volumes of interest by FSC. The script can be run by typing the following text in the command line:

python determine_relative_pixel_size.py --ref_map map1.mrc --angpix_ref_map 1.40 --map map2.mrc --angpix_map_nominal 1.36

The result appears as follows:

BEST:1.282 range:1.274 - 1.29

The precision that can be obtained depends on several factors. If one uses the script, and therefore *relion_image_handler*, one limitation is the rescaling, since *relion_image_handler* uses padding/cropping in reciprocal space. This is dependent on the box size, which means that the accuracy is inversely dependent on the box size. For a 100-pixel box size, the precision will be 2% of the pixel size (0.02 Å for a pixel size of 1 Å per pixel). With a 200-pixel box size it will be 1% of the pixel size. In our tests, we observed that the cross-correlation shows a finer sampling using *Chimera* than using FSC measurements in combination with rescaling of the maps. Therefore, the pixel size and range determined by the script serve as a starting point and boundaries for the next fitting in *Chimera*. Any pixel values within the calculated range can be used to fit the maps in *Chimera* until the cross-correlation is optimized (‘Fit to Map’) [Fig. 3 ▸(*a*)]. The cross-correlation of the initial and final maps can also be plotted using FSC in *RELION*:

relion_image_handler --i map1.mrc --fsc map2_rescaled.mrc --angpix 1.40 > fsc.star

The correlation between the maps increases when an optimal scaling factor is found [Fig. 3 ▸(*b*)]. In our particular example, the relative pixel size was off by approximately 6% (actual, 1.28 Å per pixel; nominal, 1.36 Å per pixel) and the calculated scaling factor was 0.914 (actual, 1.28 Å per pixel; reference, 1.40 Å per pixel).

It should be noted that to perform this cross-correlation in *RELION* successfully, the origin of the maps to be compared needs to be the same. The reason is that after rescaling, the origin of the rescaled map will be shifted. This can be adjusted using the *Chimera* ‘Fit in map’ option in combination with the vop resample command. Firstly, the resampled map needs to be superposed as described above to fit its coordinates to those of the map with which it should be compared. Secondly, by using the vop resample command, a new map with the new coordinates can be saved (vop resample #0 onGrid #1, where #0 is map2_rescaled.mrc and #1 is map1.mrc) and used for FSC calculations.

### 2.3. Merging data sets   

There are different methods of combining cryo-EM data sets with different pixel sizes. One can rescale the original micrographs (Method 1) or rescale the extracted particles (Method 2) (Figs. 1 ▸ and 2 ▸). Each has specific advantages and disadvantages in handling. For instance, Method 1 involves reprocessing of the data sets after rescaling the micrographs, while in Method 2 rescaling is performed at the level of particles and the user does not have to reprocess raw movies. On the other hand, Method 1 requires less manipulation of the files after merging when compared with Method 2. In either case, the final result should be similar. For both methods, since rare 2D views might go missing with extensive 2D classification before combining the data, it is recommended to merge unclassified particles.

#### 2.3.1. Method 1   

**Figure 4:**
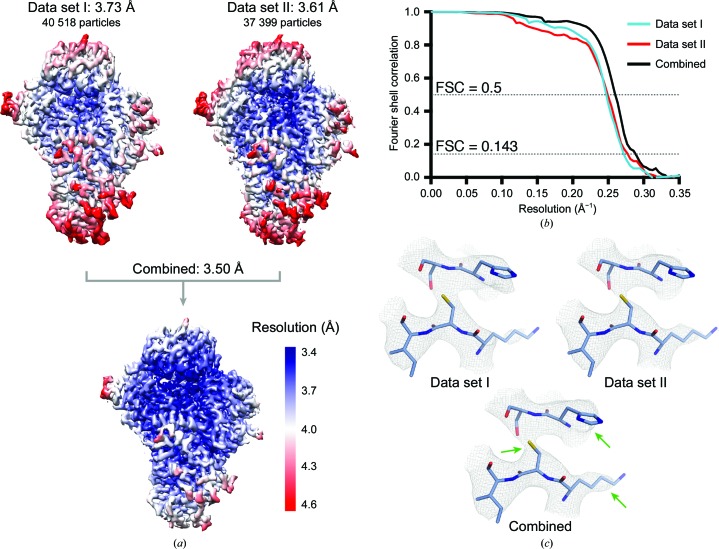
Merging data sets of the polymerase module of CPF from yeast using Method 1. (*a*) Reconstructions of the polymerase module of CPF are shown before and after joining the data. The global and local resolution of the final 3D structure (EMDB-3908) improves after combining the data sets. Maps from data sets I and II represent the particle contribution of each data set to the final structure. Local resolution was determined using *RELION* for 3D reconstructions of particles from data sets I and II alone (at the final/scaled pixel size, *i.e.* 1.40 Å per pixel) and from combining data sets after scaling data set II using a relative pixel size of 1.28 Å per pixel. Resolutions are given according to the gold-standard criteria. (*b*) Fourier shell correlation plots for gold-standard refinements. (*c*) Example density for data sets I and II and combined data sets. The final 3D structure shows finer details in the main chain and side chains when compared with the individual data sets, as indicated by green arrows.

In this example, with the same CPF data sets as described above, large-scale data acquisition (4227 movies) was required to overcome preferred orientation (Supplementary Fig. S1). Because microscope time was limited, we opted to collect data using independent microscopes. Initial data processing in *RELION* 2.0 (Fernandez-Leiro & Scheres, 2017 ▸) yielded 3D maps to a resolution of approximately 4.00 Å from each microscope. The published structure (EMDB-3908) was obtained by combining 333 550 particles and 126 617 particles from data set I and data set II, respectively. After merging and further processing of the data, we obtained a final 3D reconstruction at 3.50 Å resolution with a total of 77 917 particles (17% of the total particles), with a contribution of 40 518 particles from data set I (3.73 Å) and 37 399 particles from data set II (3.61 Å) (Fig. 4 ▸ and Supplementary Fig. S1).

(i) *Rescale micrographs.* The original micrographs can be rescaled using *relion_image_handler*. To do this, one can use the previously determined relative pixel size (1.28 Å per pixel) to rescale the micrographs of the data set II to the final required pixel size (1.40 Å per pixel). Type in the command line:

relion_image_handler --i old_mics_dataset2.mrc --o rescaled_mics_dataset2.mrc --angpix 1.28 --rescale angpix 1.40

Rescaling can be performed on average micrographs (.mrc) and movies (.mrcs). Note that to perform a later polishing step (recommended), it is necessary to rescale the movies. The advantage of rescaling both average micrographs and movies is that the motion-correction step, which is computationally intensive, does not need to be performed again.

(ii) *Rerun CTF estimation.* CTF parameters need to be recalculated for the scaled micrographs using their new true pixel size (1.40 Å per pixel). Inaccuracies in the nominal pixel size affect both defocus and contrast transfer function (CTF) estimations (Zhu *et al.*, 1997 ▸). As the CTF causes resolution-dependent amplitude modulations and phase reversals in the image, an accurate estimate of CTF parameters is essential to determine 3D structures, particularly at high resolution, when phase shifts become more relevant (Mindell & Grigorieff, 2003 ▸; Cheng *et al.*, 2015 ▸). Running CTF estimation on the newly rescaled data set is computationally cheap, and it will guarantee the accuracy of the CTF. After this step, the data sets can be treated as if they had been recorded on the same microscope with the same magnification and pixel size. Particle picking, extraction, 2D classification and further data processing can be performed as before. There is no need to calculate a new box size, and data sets can be merged using the ‘Join STAR files’ job in *RELION* (Fig. 2 ▸; Fernandez-Leiro & Scheres, 2017 ▸).

(iii) *Saving time by rescaling coordinates of previously picked particles.* One can save processing or manual picking time by rescaling the coordinates of the already picked or extracted particles from the initial processing. The coordinates will have changed owing to the rescaling of the micrographs. In addition, the magnification and the path to the micrographs will need to be updated in the STAR file. We provide a second Python script called rescale_particles.py (Supplementary Script 2) that takes several columns from a particles.star/data.star file and corrects them based on the pixel sizes. The columns that it uses are _rlnMicrographName, _rlnCoordinateX, _rlnCoordinateY, _rlnOriginX, _rlnOriginY, _rlnMagnification and _rlnDetectorPixelSize. The inputs for the script are the nominal pixel size used for the *RELION* run, the relative pixel size calculated and the target/rescaled pixel size. It also allows the name of the micrographs in the STAR file to be changed (*e.g.* a new path where the rescaled MRC files are located). The output file is ready to be imported into *RELION* (‘Import’) and can be readily used.

python rescale_particles.py --i dataset2.star --o dataset2_rescale.star --pix_nominal 1.36 --pix_relative 1.28 -pix_target 1.40 --mrc_name_path MRCS_dataset2/

Using this methodology, we successfully combined data sets of the 200 kDa polymerase module of CPF and obtained an improved 3D reconstruction at 3.50 Å resolution. The final map shows an overall improvement in local resolution and density quality (Fig. 4 ▸; Casañal *et al.*, 2017 ▸).

#### 2.3.2. Method 2   

**Figure 5:**
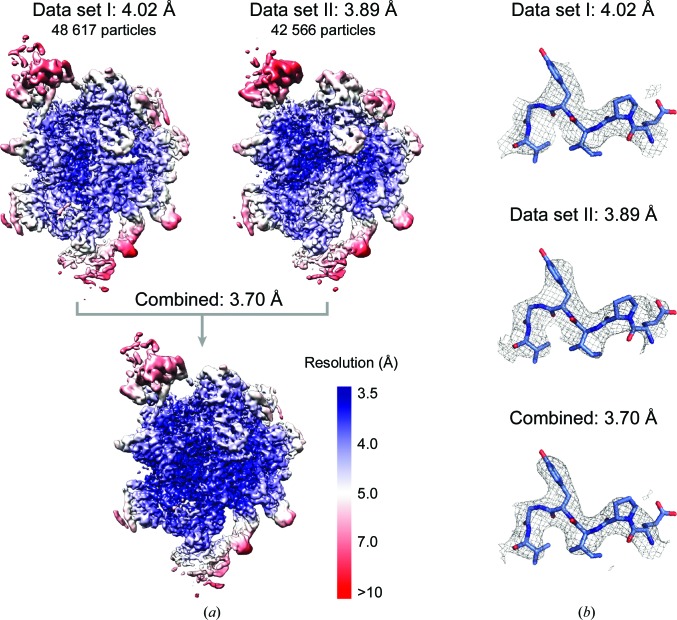
Merging data sets of the yeast post-catalytic (P complex) spliceosome using Method 2. (*a*) The global and local resolution of the final 3D structure improves after combining the data sets. Local resolution was determined using *RELION* for 3D reconstructions of particles from data sets I and II alone (at the final/scaled pixel size) and from combining data sets after scaling data set II using a relative pixel size of 0.880 Å per pixel. Resolutions are given according to the gold-standard criteria. (*b*) Example density for data sets I and II and combined data sets.

**Figure 6:**
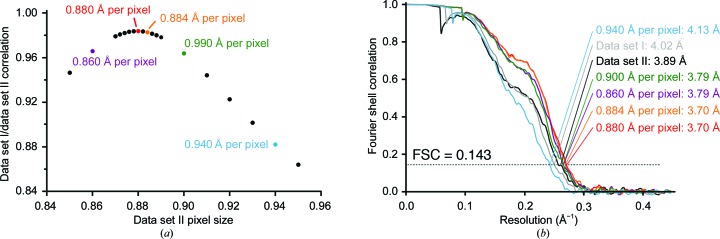
Effect of scaling. (*a*) Correlation between the 3D reconstructions calculated from data set I and data set II (at 1.120 Å per pixel) using various pixel sizes for data set II. The correlation was calculated using *Chimera* as described in the text. (*b*) Gold-standard FSC for spliceosome reconstructions for data set I alone (light grey), data set II alone (black) and from combining data as described in the text (red). Additional curves come from mis-scaling data set II using the indicated pixel size to 1.120 Å per pixel, then merging with data set I, refining and post-processing. These curves are coloured the same as the corresponding points in (*a*). Resolutions are given according to the gold-standard criteria.

Method 2 differs from Method 1 by not scaling micrographs, but rather scaling particles during extraction from the micrographs (Figs. 1 ▸ and 2 ▸). This method is demonstrated using the structure of the yeast post-catalytic spliceosome captured immediately after the exon-ligation reaction (Wilkinson *et al.*, 2017 ▸). The published structure (EMDB-3979) was obtained from a single data set of 48 617 particles collected at 1.120 Å per pixel to 3.73 Å resolution. Subsequently, a second data set of 42 566 particles was collected at 0.900 Å per pixel, which refined to 3.89 Å resolution (Supplementary Figs. S1 and S2). Both data sets were collected on Krios 1 at MRC-LMB. The original data set (larger pixel size) is referred to as ‘data set I’, and in this example unpolished particles were used, which refined to 4.02 Å resolution (Fig. 5 ▸). The relative pixel size of data set II was obtained using *Chimera*, as detailed above, which produced a clear peak of cross-correlation to data set I at 0.880 Å per pixel (Fig. 6 ▸).

(i) *Re-calculate CTF defocus values*. In the example here, data set II was originally processed (including CTF estimation) using a pixel size of 0.900 Å per pixel, which was the reported nominal pixel size at the magnification of data collection. After the determination of the relative pixel size (0.880 Å), the CTF parameters need to be re-estimated considering the new pixel size. Alternatively, since to a good approximation the fitted defocus values will depend on the square of the pixel size, one could just multiply these values by (0.880/0.900)^2^:

awk ‘NF < 5 {print $0}; /mrc/{$3 = $3*(0.880/0.900)**2;$4=$4*(0.880/0.900)**2; $10=10000; $11=0.880 print $0}’ micrographs_ctf.star > micrographs_ctf_newapix.star

In this example, $3 and $4 refer to columns 3 and 4 of the STAR file, which usually correspond to _rlnDefocusU and _rlnDefocusV, $10 and $11 refer to columns 10 and 11, corresponding to _rlnMagnification and _rlnDetectorPixelSize, and 0.880/0.900 needs to be substituted for the correct ratio between the relative (new) pixel size and the nominal (old) pixel size. The correct column numbers can be found by looking at the first few lines of the *RELION* STAR file. Something similar can also be achieved with the supplied bash script scale_ctf.sh (Supplementary Script 3), which additionally applies a small constant correction to somewhat account for higher order terms in the CTF equations. It is run as follows (where bold indicates user input):

**scale_ctf.sh micrographs_ctf.star**Starting apix: **0.900**New apix: **0.880**Read spherical aberration as 27000000 A from star-file headerAcceleration voltage read as 300, will use electron wavelength of 0.0197 ADefocus scaled by 0.956049 plus a constant correction of -14.602555Wrote out micrographs_ctf_newapix.star

Besides the defocus values, errors in the pixel size also impact the spherical aberration (Cs) term. While the defocus is directly proportional to the square of the wavenumber, the Cs is proportional to the fourth power of the wavenumber. Therefore, the defocus term dominates at lower resolutions, but at higher resolutions the Cs becomes significant. The Cs can be scaled [*e.g.* for a Cs of 2.7 mm, the new Cs is 2.7 × (0.900/0.880)^4^] and modified in the STAR file. With *CtfRefine* one can keep Cs as it is and refine only defocus values. The fit then becomes accurate.

(ii) *Re-extract and scale particles*. In *RELION* a convenient way to scale particles is during the ‘Particle Extraction’ job type. In this job, one has the option of extracting at a particular box size and scaling this box to another box size. (If one no longer has the micrographs on disk or wishes to scale polished particles, one can also scale extracted particles using *relion_preprocess*). A restriction is that the box sizes must be even-numbered. In the example given, data set II is rescaled from 0.880 Å per pixel to 1.120 Å per pixel and has a final box size of 420 pixels (the box size of data set I). To scale straight to a box of 420 pixels, one would have to extract with a box size of 420 × 1.120/0.880 = 534.5 pixels, which is not possible. A possibility would be to extract with a box of 534 pixels and scale to 420 pixels, giving a scaled pixel size of 534/420 × 0.880 = 1.119, which might be accurate enough (see below). Alternatively, we provide a Python script boxscaler.py (Supplementary Script 4), which can find a pair of even-numbered box sizes very close to the desired ratio. It is run as follows (bold indicates user input):

**boxscaler.py**What is the smallest box size to search from? **420**What is the largest box size to search to? **600**What is your starting pixel size? **0.880**and how many answers do you want? **1**Start with a 560 pixel box, scale to a 440 pixel boxThis will give a scaling factor of 0.78571, compared to a desired pixel size ratio of 0.78571, giving a 0.000 per cent error.

This script shows that extracting at box size 560 and scaling to box size 440 will give the desired scaling factor to 0% error. One can perform this in *RELION* by creating a ‘Particle extraction’ job using the run_data.star file generated from refinement of data set II to provide the particle coordinates (‘Refined particles STAR file’ field) and using the micrographs_ctf_newapix.star file generated in step 1 of Method 2 to provide the micrographs with the correct CTF parameters (‘micrograph STAR file’ field). ‘Particle box size’ need to be specified as 560 pixels, ‘Rescale particles’ as ‘Yes’ and ‘Re-scaled size’ as 440 pixels. CRITICAL: from *RELION* v.3 onwards (Zivanov *et al.*, 2018 ▸), if a run_data.star file is used for extraction, CTF parameters are by default taken from this file. Add the --use_ctf_in_mic flag to ‘Additional arguments’ to make sure that CTF parameters are taken from micrographs_ctf_newapix.star. Manually specify the diameter of the background circle for particle normalization as if we were extracting with a box size of 420. For example, the default diameter is 75% of the box size before scaling. Here, the new diameter of the background circle should be 400 pixels = 0.75 × 420 × 1.120/0.880.

(iii) *Crop particles to the correct box size*. Particles are now on the correct scale but with a box size of 440 (data set II), and need to be merged with particles with a box size of 420 (data set I). One can crop to a box size of 420 using* relion_preprocess* as follows:

relion_preprocess --operate_on Extract/job043/particles_star --window 420 --operate_out dataset2_particles_scaled_window420

This will write out a stack of particles dataset2_particles_scaled_window420.mrcs and a corresponding STAR file dataset2_particles_scaled_window420.star. This STAR file can now be merged with the data set I STAR file using the ‘Join STAR files’ job in *RELION*.

In this example, combining the data sets gave a reconstruction at 3.70 Å resolution, with significant improvement in local resolution and density quality compared with either data set reconstructed individually (Fig. 5 ▸). After further processing using CTF refinement and Bayesian polishing in *RELION* 3.0 (Zivanov *et al.*, 2018 ▸), we were able to improve the resolution to 3.30 Å, a significant improvement over the published reconstruction (Supplementary Fig. S2).

Note: to integrate this method with Bayesian polishing, perform polishing on data set II supplying the following additional flags under ‘additional arguments’: --window 560 --scale 440

As a result, polished particles will be produced at the correct size but with a box size of 440. These can be cropped as above as follows:

relion_preprocess --operate_on Polish/job043/shiny.star --window 420 --operate_out dataset2_particles_shiny_scaled

The output will be a particle stack dataset2_particles_shiny_scaled.mrcs and a STAR file dataset2_particles_shiny_scaled.star, which can be joined with data set I.

## 3. Effect of pixel size on resolution   

For several reasons, it may not be possible to precisely calculate the correct scaling factor. For example, the resolution of the individual maps may not be high enough for a discrete peak of correlation to emerge when aligning maps in *Chimera*, or a convenient ratio of box sizes that gives the correct scaling factor may not be available. We tested how accurately the scaling factor needs to be determined (Fig. 6 ▸). We scaled data set II in the spliceosome example using different starting pixel sizes: the correct 0.880 Å per pixel, the almost correct 0.884 Å per pixel, the incorrect 0.940 Å per pixel and two close sizes of 0.860 and 0.900 Å per pixel [Fig. 6 ▸(*a*)]. For each case we recalculated the CTF parameters using *Gctf* (Zhang, 2016 ▸) and extracted with a 560-pixel box, scaled to various box sizes (*e.g.* 440 pixels for 0.880 Å per pixel, 470 pixels for 0.940 Å per pixel) and cropped to 420 pixels. After refinement, we determined the resolution using the same mask [Fig. 6 ▸(*b*)]. We found that using 0.884 Å per pixel gave a reconstruction that was almost identical to the correct 0.880 Å per pixel. Either 0.860 or 0.900 Å per pixel gave only a small reduction in resolution, while 0.940 Å per pixel gave a reconstruction with worse resolution than either of the starting data sets alone [Fig. 6 ▸(*b*)]. This analysis shows that the resolution obtained after reconstruction from merged data sets is relatively tolerant to scaling-factor error, at least in the 3.70 Å resolution range for a 1–2 MDa particle: 0.5–2% error is acceptable, while 7% error is not. In principle, higher accuracy should be required when analysing a larger complex or a higher resolution structure (although both of these factors should also facilitate more accurate scaling-factor determination).

## 4. Conclusion   

In cryo-EM, protein samples often require extensive biochemical optimization, including sample preparation and vitrification. To obtain structural information that helps to gain insight into specific biological problems, researchers often acquire large data sets or collect data from different preparations. Merging data sets recorded at different microscopes, or with varying conditions of imaging, therefore becomes an important task. In this report, we describe two methods (scaling micrographs or particles) to combine cryo-EM data collected at different pixel sizes successfully. We have also shown how errors in pixel-size determination correlate with resolution and provide scripts for the accurate determination of pixel sizes. In our examples, data sets from the same type of microscope (Titan Krios) and detector (K2 equipped with a GIF energy filter) have been merged, improving the resolution. This methodology can be further extended to other cases in which cryo-EM data sets have been acquired with different types of microscopes and detectors. When combining data collected using different detectors, each detector will have a specific MTF file. Different MTF files are likely to have a minimal effect in the final 3D reconstruction, but further analysis is necessary to report their impact at near-Nyquist resolution. It should be noted that adding more data to existing data sets is not always a means to improve the quality of 3D reconstructions. For example, when data quality limits resolution, additional particles will have a marginal effect. To decide whether further data collection is useful, a good strategy is to merge the existing data sets one by one (starting with the best). This will determine whether combining more particles of similar quality improves the quality of the 3D reconstruction. To estimate the number of particles (of similar quality) required to increase resolution one can use Rosenthal and Henderson plots (Rosenthal & Henderson, 2003 ▸; Zivanov *et al.*, 2018 ▸). In our examples, the addition of about 50% more particles helped to increase the resolution of the 3D reconstructions from 3.61 to 3.50 Å for the polymerase module of CPF, and the addition of 100% more particles increased the resolution from 3.89 to 3.70 Å for the post-catalytic (P complex) spliceosome. Importantly, improving the quality of the data will also improve the resolution of the resulting 3D reconstruction (Naydenova & Russo, 2017 ▸). For example, the correction of beam tilt using the new tools in *RELION* 3.0 (Zivanov *et al.*, 2018 ▸) improves the resolution of the polymerase module from data set I to a greater extent than merging with data set II.

We expect that merging data sets will become more relevant in the future as more challenging cryo-EM projects are tackled where one data-collection session is not enough. This work contributes to making merging data sets a routine job.

## 5. Related literature   

The following references are cited in the supporting information for this article: Scheres (2014 ▸), Zhang (2016 ▸) and Zheng *et al.* (2017 ▸).

## Supplementary Material

Script 1: determine__relative_pixel_size.py. DOI: 10.1107/S2059798319010519/ic5109sup1.exe

Script 2: rescale_particles.py. DOI: 10.1107/S2059798319010519/ic5109sup2.exe

Script 3: scale_ctf.sh. DOI: 10.1107/S2059798319010519/ic5109sup3.exe

Script 4: boxscaler.py. DOI: 10.1107/S2059798319010519/ic5109sup4.exe

Supplementary methods, data and figures. DOI: 10.1107/S2059798319010519/ic5109sup5.pdf

## References

[bb1] Ashtiani, D., Venugopal, H., Belousoff, M., Spicer, B., Mak, J., Neild, A. & de Marco, A. (2018). *J. Struct. Biol.* **203**, 94–101. 10.1016/j.jsb.2018.03.012 29630922

[bb2] Battaglia, M., Contarato, D., Denes, P., Doering, D., Giubilato, P., Kim, T. S., Mattiazzo, S., Radmilovic, V. & Zalusky, S. (2009). *Nucl. Instrum. Methods Phys. Res. A*, **598**, 642–649.

[bb3] Boland, A., Martin, T. G., Zhang, Z., Yang, J., Bai, X.-C., Chang, L., Scheres, S. H. W. & Barford, D. (2017). *Nature Struct. Mol. Biol.* **24**, 414–418. 10.1038/nsmb.3386 PMC5385133 28263324

[bb4] Casañal, A., Kumar, A., Hill, C. H., Easter, A. D., Emsley, P., Degliesposti, G., Gordiyenko, Y., Santhanam, B., Wolf, J., Wiederhold, K., Dornan, G. L., Skehel, M., Robinson, C. V. & Passmore, L. A. (2017). *Science*, **358**, 1056–1059. 10.1126/science.aao6535 PMC5788269 29074584

[bb5] Charenton, C., Wilkinson, M. E. & Nagai, K. (2019). *Science*, **364**, 362–367. 10.1126/science.aax3289 PMC6525098 30975767

[bb6] Chen, J., Noble, A. J., Kang, J. Y. & Darst, S. A. (2019). *J. Struct. Biol. X.*, 100005. 10.1016/j.yjsbx.2019.100005 PMC7153306 32285040

[bb7] Cheng, Y., Glaeser, R. M. & Nogales, E. (2017). *Cell*, **171**, 1229–1231. 10.1016/j.cell.2017.11.016 PMC6186021 29195065

[bb8] Cheng, Y., Grigorieff, N., Penczek, P. A. & Walz, T. (2015). *Cell*, **161**, 438–449. 10.1016/j.cell.2015.03.050 PMC4409659 25910204

[bb9] Cianfrocco, M. A., Lahiri, I., DiMaio, F. & Leschziner, A. E. (2018). *J. Struct. Biol.* **203**, 230–235. 10.1016/j.jsb.2018.05.014 PMC6091888 29864529

[bb10] Drulyte, I., Johnson, R. M., Hesketh, E. L., Hurdiss, D. L., Scarff, C. A., Porav, S. A., Ranson, N. A., Muench, S. P. & Thompson, R. F. (2018). *Acta Cryst.* D**74**, 560–571. 10.1107/S2059798318006496 PMC6096488 29872006

[bb11] Faruqi, A. R. & McMullan, G. (2011). *Q. Rev. Biophys.* **44**, 357–390. 10.1017/S0033583511000035 21524337

[bb12] Fernández, I. S., Bai, X.-C., Hussain, T., Kelley, A. C., Lorsch, J. R., Ramakrishnan, V. & Scheres, S. H. W. (2013). *Science*, **342**, 1240585. 10.1126/science.1240585 PMC3836175 24200810

[bb13] Fernandez-Leiro, R. & Scheres, S. H. W. (2017). *Acta Cryst.* D**73**, 496–502. 10.1107/S2059798316019276 PMC5458491 28580911

[bb14] Fica, S. M., Oubridge, C., Wilkinson, M. E., Newman, A. J. & Nagai, K. (2019). *Science*, **363**, 710–714. 10.1126/science.aaw5569 PMC6386133 30705154

[bb15] Guo, H., Suzuki, T. & Rubinstein, J. L. (2019). *Elife*, **8**, e43128. 10.7554/eLife.43128 PMC6377231 30724163

[bb16] Harauz, G. & van Heel, M. (1986). *Optik*, **73**, 146–156.

[bb17] Kimanius, D., Forsberg, B. O., Scheres, S. H. W. & Lindahl, E. (2016). *Elife*, **5**, 19. 10.7554/eLife.18722 PMC5310839 27845625

[bb18] Kühlbrandt, W. (2014). *Science*, **343**, 1443–1444. 10.1126/science.1251652 24675944

[bb19] Li, X., Zheng, S. Q., Egami, K., Agard, D. A. & Cheng, Y. (2013). *J. Struct. Biol.* **184**, 251–260. 10.1016/j.jsb.2013.08.005 PMC3854003 23968652

[bb20] Liu, N., Zhang, J., Chen, Y., Liu, C., Zhang, X., Xu, K., Wen, J., Luo, Z., Chen, S., Gao, P., Jia, K., Liu, Z., Peng, H. & Wang, H. W. (2019). *J. Am. Chem. Soc.* **141**, 4016–4025. 10.1021/jacs.8b13038 30724081

[bb21] Matzov, D., Aibara, S., Basu, A., Zimmerman, E., Bashan, A., Yap, M.-N. F., Amunts, A. & Yonath, A. E. (2017). *Nature Commun.* **8**, 723. 10.1038/s41467-017-00753-8 PMC5620080 28959035

[bb22] McMullan, G., Faruqi, A. R., Clare, D. & Henderson, R. (2014). *Ultramicroscopy*, **147**, 156–163. 10.1016/j.ultramic.2014.08.002 PMC4199116 25194828

[bb23] Menny, A., Serna, M., Boyd, C. M., Gardner, S., Joseph, A. P., Morgan, B. P., Topf, M., Brooks, N. J. & Bubeck, D. (2018). *Nature Commun.* **9**, 5316. 10.1038/s41467-018-07653-5 PMC6294249 30552328

[bb24] Meyerson, J. R., Rao, P., Kumar, J., Chittori, S., Banerjee, S., Pierson, J., Mayer, M. L. & Subramaniam, S. (2014). *Sci. Rep.* **4**, 7084. 10.1038/srep07084 PMC4235105 25403871

[bb25] Mindell, J. A. & Grigorieff, N. (2003). *J. Struct. Biol.* **142**, 334–347. 10.1016/s1047-8477(03)00069-8 12781660

[bb26] Naydenova, K. & Russo, C. J. (2017). *Nature Commun.* **8**, 629. 10.1038/s41467-017-00782-3 PMC5607000 28931821

[bb27] Noble, A. J., Dandey, V. P., Wei, H., Braschi, J., Chase, J., Acharya, P., Tan, Y. Z., Zhang, Z., Kim, L. Y., Scapin, G., Rapp, M., Eng, E. T., Rice, W. J., Cheng, A., Negro, C. J., Shapiro, L., Kwong, P. D., Jeruzalmi, D., des Georges, A., Potter, C. S. & Carragher, B. (2018). *Elife*, **7**, e34257. 10.7554/eLife.34257 PMC5999397 29809143

[bb28] Noble, A. J., Wei, H., Dandey, V. P., Zhang, Z., Tan, Y. Z., Potter, C. S. & Carragher, B. (2018). *Nature Methods*, **15**, 793–795. 10.1038/s41592-018-0139-3 PMC6168394 30250056

[bb29] Nogales, E., Louder, R. K. & He, Y. (2016). *Curr. Opin. Struct. Biol.* **40**, 120–127. 10.1016/j.sbi.2016.09.009 PMC5161697 27689812

[bb30] Pettersen, E. F., Goddard, T. D., Huang, C. C., Couch, G. S., Greenblatt, D. M., Meng, E. C. & Ferrin, T. E. (2004). *J. Comput. Chem.* **25**, 1605–1612. 10.1002/jcc.20084 15264254

[bb31] Plaschka, C., Lin, P.-C. & Nagai, K. (2017). *Nature (London)*, **546**, 617–621. 10.1038/nature22799 PMC5503131 28530653

[bb32] Rosenthal, P. B. & Henderson, R. (2003). *J. Mol. Biol.* **333**, 721–745. 10.1016/j.jmb.2003.07.013 14568533

[bb33] Russo, C. J. & Passmore, L. A. (2014). *Science*, **346**, 1377–1380. 10.1126/science.1259530 PMC4296556 25504723

[bb34] Russo, C. J. & Passmore, L. A. (2016). *Curr. Opin. Struct. Biol.* **37**, 81–89. 10.1016/j.sbi.2015.12.007 PMC4863039 26774849

[bb35] Scheres, S. H. W. (2012). *J. Struct. Biol.* **180**, 519–530. 10.1016/j.jsb.2012.09.006 PMC3690530 23000701

[bb50] Scheres, S. H. W. (2014). *Elife*, **3**, e03665. 10.7554/eLife.03665 PMC4130160 25122622

[bb36] Scheres, S. H. W. & Carazo, J.-M. (2009). *Acta Cryst.* D**65**, 672–678. 10.1107/S0907444909012049 PMC2703573 19564687

[bb37] Smith, M. T. J. & Rubinstein, J. L. (2014). *Science*, **345**, 617–619. 10.1126/science.1256358 25104368

[bb38] Takizawa, Y., Binshtein, E., Erwin, A. L., Pyburn, T. M., Mittendorf, K. F. & Ohi, M. D. (2017). *Protein Sci.* **26**, 69–81. 10.1002/pro.3054 PMC5192976 27673321

[bb39] Urnavicius, L., Zhang, K., Diamant, A. G., Motz, C., Schlager, M. A., Yu, M., Patel, N. A., Robinson, C. V. & Carter, A. P. (2015). *Science*, **347**, 1441–1446. 10.1126/science.aaa4080 PMC4413427 25814576

[bb40] Wade, R. H. (1992). *Ultramicroscopy*, **46**, 145–156.

[bb41] Wilkinson, M. E., Fica, S. M., Galej, W. P., Norman, C. M., Newman, A. J. & Nagai, K. (2017). *Science*, **358**, 1283–1288. 10.1126/science.aar3729 PMC5808836 29146871

[bb42] Wilkinson, M. E., Lin, P.-C., Plaschka, C. & Nagai, K. (2018). *Annu. Rev. Biophys.* **47**, 175–199. 10.1146/annurev-biophys-070317-033410 29494253

[bb43] Zhang, K. (2016). *J. Struct. Biol.* **193**, 1–12. 10.1016/j.jsb.2015.11.003 PMC4711343 26592709

[bb52] Zheng, S. Q., Palovcak, E., Armache, J.-P., Verba, K. A., Cheng, Y. & Agard, D. A. (2017). *Nature Methods*, **14**, 331–332. 10.1038/nmeth.4193 PMC5494038 28250466

[bb44] Zhu, J., Penczek, P. A., Schröder, R. & Frank, J. (1997). *J. Struct. Biol.* **118**, 197–219. 10.1006/jsbi.1997.3845 9169230

[bb45] Zivanov, J., Nakane, T., Forsberg, B. O., Kimanius, D., Hagen, W. J., Lindahl, E. & Scheres, S. H. W. (2018). *Elife*, **7**, 163. 10.7554/eLife.42166 PMC6250425 30412051

[bb46] Zivanov, J., Nakane, T. & Scheres, S. H. W. (2019). *IUCrJ*, **6**, 5–17. 10.1107/S205225251801463X PMC6327179 30713699

