# Supplementary material for: Methods for merging data sets in electron cryo-microscopy
Source: Acta Crystallogr D Struct Biol. 2019 Aug 23;75(Pt 9):782–91. doi: 10.1107/S2059798319010519 (PMC6719665; doi:10.1107/S2059798319010519)
Supplement: Supplementary file 5 [file d-75-00782-sup5.pdf]

STRUCTURAL
BIOLOGY

Volume 75 (2019)

Supporting information for article:

Methods for merging data sets in electron cryo-microscopy

Max E. Wilkinson, Ananthanarayanan Kumar and Ana Casañal

S1. Supplementary Methods

S1.1. Data processing workflow of the P-complex spliceosome

The main text describes for the P-complex spliceosome a simplified version of the following data-processing workflow that yielded a final reconstruction at 3.30 Å, compared to the originally published 3.70 Å reconstruction (Supplementary Figure 2, S2).

Dataset I referred to in the main text is described in (Wilkinson et al., 2017) and corresponds to the particles with a docked 3' splice site. For dataset II, a grid prepared under the same condition as dataset I was imaged in an FEI Titan Krios transmission electron microscope operated in EFTEM mode at 300 kV using the Gatan K2 Summit direct electron detector and a GIF Quantum energy filter (slit width 20 eV). Micrographs were collected automatically using EPU, collecting 1,441 movies at a nominal magnification of 130,000x (0.88 Å/px). The camera was operated in counting mode with a total exposure time of 7 s fractionated into 35 frames and a total dose of 45 e^{-}Å^{-2} per movie. During the same session 173 movies were collected in super-resolution mode (0.44 Å/px); particles from these movies were not included in the example in the main text for simplicity, but were included in the final reconstruction. Movies were corrected for movement using MotionCor2 (Zheng et al., 2017), applying 5 × 5 patching and applying dose-weighting to individual frames. The super-resolution micrographs were binned by 2, then merged with the counting micrographs and processed together. CTF parameters were estimated using Gctf (Zhang, 2016) and micrographs were manually screened. Particles were picked with templates using Gautomatch as in (Wilkinson et al., 2017), yielding 79,509 particles. 3D classification with a P complex reference (EMD-3979) resulted in 48,024 good particles that after polishing in *RELION 2.0* (Scheres, 2014) were refined to 3.56 Å resolution. These were scaled to 1.12 Å/px as described in the main text, and merged with dataset I. Another round of 3D classification resulted in 52,748 particles in the 3'SS-docked state that refined to 3.50 Å resolution. After CTF refinement in *RELION 3.0* (Zivanov et al., 2019) the resolution further improved to 3.30 Å. This final map is deposited in the EMDB under accession code EMD-XXXX.

Supplementary Data

Supplementary data 1, Script 1: determine_relative_pixel_size.py

The script determine_relative_pixel size.py is designed to determine the relative pixel size of one map (--map, --angpix_map_nominal) in relation to a reference map (--ref_map, --angpix_ref_map) using relion_image_handler. For this to work, both maps need to be in the same orientation. This script will perform density centring but will not rotate the map in any way. To align the maps before running the script, one can use the VOP command in Chimera.

To determine the relative pixel size, this script will rescale the map, around the initial nominal pixel size, centre it, and then compare it with the reference map by FSC. The pixel size will be changed in smaller and smaller intervals until a minimum is found.

Supplementary data 2, Script 2: rescale_particles.py

The script rescale_particles.py takes the particle coordinates from an input star file (--i) and writes out a star file (--o) with scaled coordinates. For this it needs the relative pixel size (--pix_relative) that the coordinates are currently at and the target pixel size (--pix_target) the coordinates should be adjusted to. With this it is possible to adjust 'rlnCoordinateX', 'rlnCoordinateY', 'rlnOriginX' and 'rlnOriginY' by simply multiplying the coordinates with the scaling factor (pix_relative/pix_target). To adjust the magnification in the star file, it is also necessary to know the nominal pixel size (--pix_nominal) that was used to get the initial star file.

In most cases, it will be necessary to adjust the file name of the micrographs. For this one can use --mrc_name_path, --mrc_name_prefix, --mrc_name_suffix, --mrc_name_replacement_in and --mrc_name_replacement_out. It is not necessary to fill out all of them, except --mrc_name_path which is necessary to set the path of the files. The name is determined by finding the last / in the path section of the star file. To add pre or suffixes to the name one can use --mrc_name_prefix and --mrc_name_suffix. --mrc_name_prefix will add the given string at the beginning of the name, --mrc_name_suffix will add the given string at the end. To replace a part of the name with another, --mrc_name_replacement_in and --mrc_name_replacement_out have to be used in combination . In this case the string to be replaced will be given in --mrc_name_replacement_in and the replacement string will be given in --mrc_name_replacement_out.

Supplementary data 3, Script 3: scale_ctf.sh

The bash and awk script scale_ctf.sh allows one to skip CTF recalculation once the relative pixel size of a data set is determined. It takes as input any STAR file containing CTF parameters, for example the output from CTF calculation or 3D refinement. The script will then prompt for the initial and desired pixel sizes, and will alter the magnification and defocus values of the STAR file. The defocus is altered by the squared ratio of the initial and desired pixel size, plus a correction determined as follows:

The phase of the CTF, γ, is determined by Eq. (1)

$$\gamma(\vec{s}) = \gamma(s,\theta) = -\frac{\pi}{2}C_s\lambda^3 s^4 + \pi\lambda z(\theta)s^2 \tag{1}$$

where $\vec{s}$ is spatial frequency represented by its modulus s and its azimuthal angle θ; C_s is the spherical aberration coefficient; λ is the electron wavelength; and $z(\theta)$ is the defocus in direction θ

Factoring out $\pi\lambda$, for a given pixel size ratio $\alpha = \frac{apix_{old}}{apix_{new}}$ and at a given angle θ we therefore desire the equality in Eq. (2)

$$\frac{-C_s\lambda^2}{2}s^4 + z_{old}s^2 = \frac{-C_s\lambda^2}{2}s^4\alpha^4 + z_{new}s^2\alpha^2 \tag{2}$$

where z_{old} is the initially calculated defocus value at angle θ and z_{new} is some new defocus value that will be consistent with $apix_{new}$

z_{new} is then given by Eq. (3)

$$z_{new} = \frac{z_{old}}{\alpha^2} + \frac{C_s\lambda^2}{2}\left(\alpha^2 - \frac{1}{\alpha^2}\right)s^2 \tag{3}$$

The first term in Eq. (3) is the simple correction of the defocus by the squared pixel size ratio. The second term depends on the spatial frequency. Empirically, we found that setting s^2 to 0.031 Å^{-2} to create a constant correction term gives similar results to CTF re-estimation using GCTF. With this value, the mean discrepancy between recalculated defocus using GCTF and using Eq. (3) is 0 Å, with a 5th to 95th percentile range of up to -40 to 40 Å. This discrepancy is negligible compared to per-particle variation in defocus, and would only have an effect on CTF phase at very high resolutions.

Supplementary data 4, Script 4: boxscaler.py

The python script boxscaler.py finds a pair of even box sizes that will produce a desired scaling factor. Running the script from the command line brings up a series of prompts asking for the starting and ending pixel sizes, the range of box sizes to search over, and the number of desired solutions. The script then calculates all possible ratios between box sizes within the range given, and finds the ratio closest to the desired scaling factor.

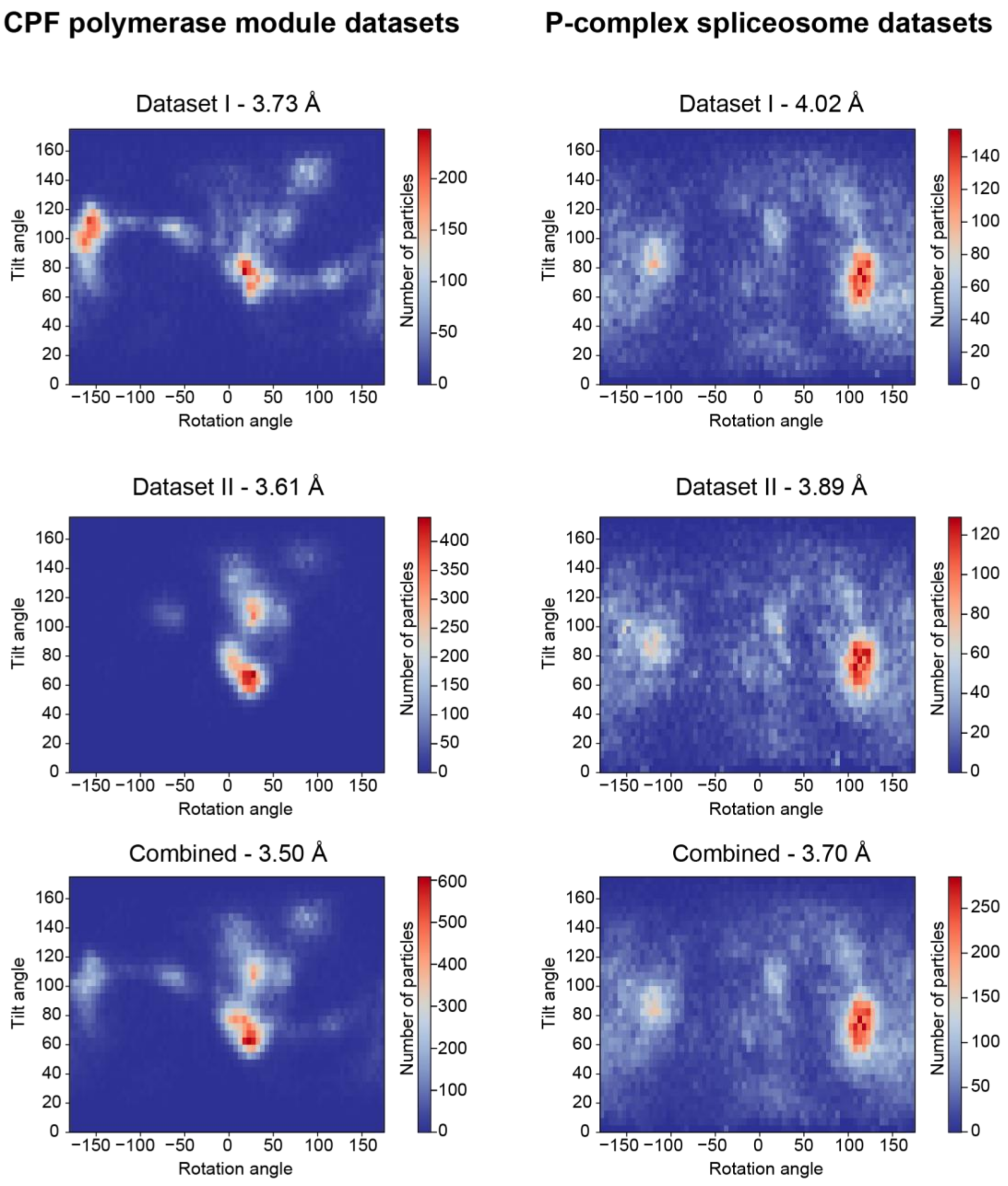

Figure S1 Orientation distribution of the polymerase module of CPF (left) and the P-complex spliceosome (right).

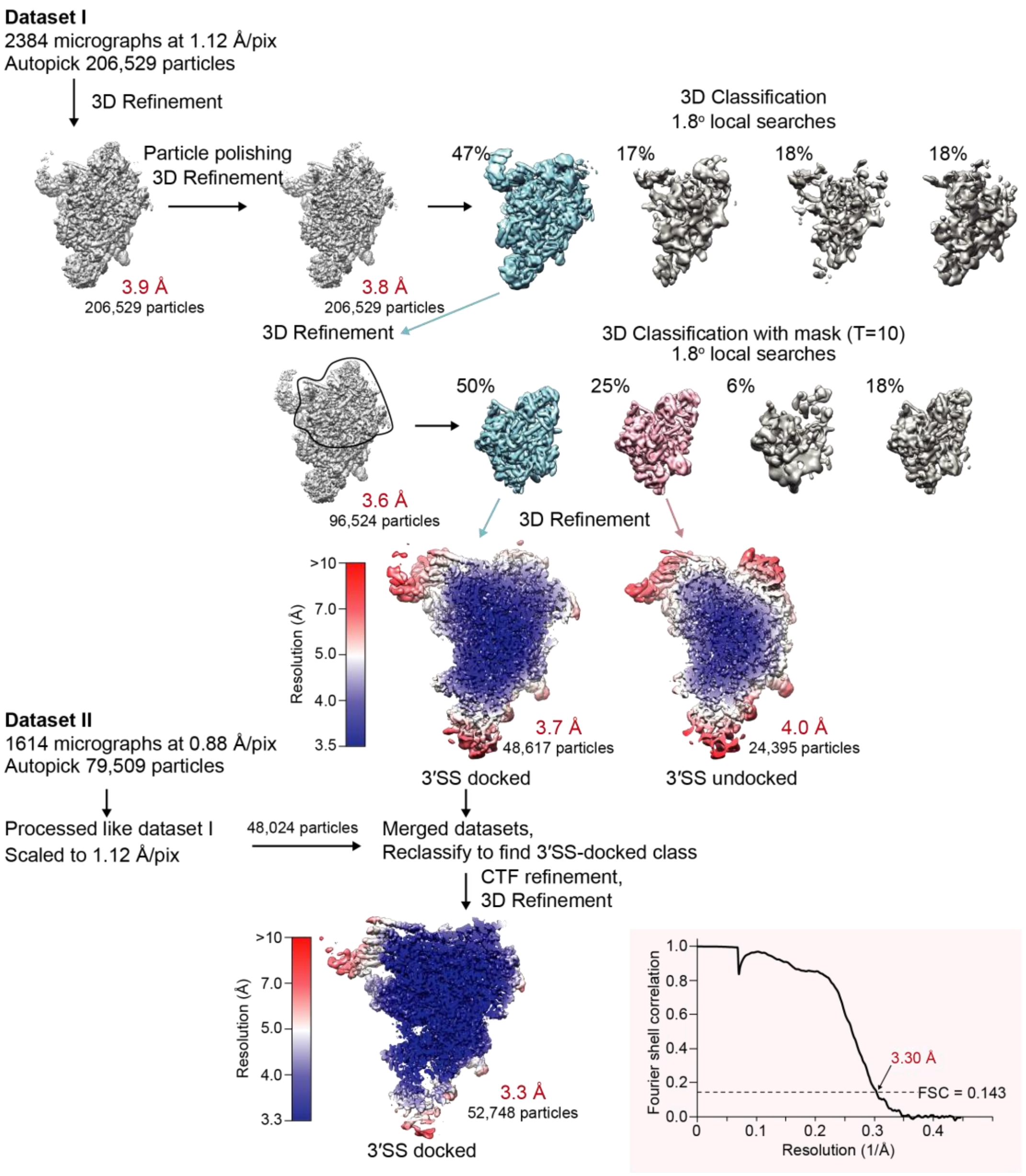

Figure S2 P-complex spliceosome cryoEM data processing. Local resolution was calculated using *RELION*. All resolution values were calculated by postprocessing using the same soft mask. The FSC curve for postprocessing the final reconstruction at 3.30 Å resolution is shown in inset (pink background). Dataset I processing flowchart adapted from Wilkinson et al., 2017.

Scheres, S. H. (2014). *Elife*. **3**, e03665.

Wilkinson, M. E., Fica, S. M., Galej, W. P., Norman, C. M., Newman, A. J., & Nagai, K. (2017). *Science*. **358**, 1283–1288.

Zivanov, J., Nakane, T., & Scheres, S. H. W. (2019). *IUCrJ. M6, 5-17*.

Zhang, K. (2016). *Journal of Structural Biology*. **193**, 1–12.

Zheng, S. Q., Palovcak, E., Armache, J. P., Verba, K. A., Cheng, Y., & Agard, D. A. (2017). *Nature Methods*. **14**, 331–332.
